# Physico-Chemical Transformation and Toxicity of Multi-Shell InP Quantum Dots under Simulated Sunlight Irradiation, in an Environmentally Realistic Scenario

**DOI:** 10.3390/nano12203703

**Published:** 2022-10-21

**Authors:** Fanny Dussert, Géraldine Sarret, Karl David Wegner, Olivier Proux, Gautier Landrot, Pierre-Henry Jouneau, Peter Reiss, Marie Carrière

**Affiliations:** 1 University Grenoble Alpes, CEA, CNRS, IRIG-SyMMES, CIBEST, 38000 Grenoble, France; 2 University Grenoble Alpes, University Savoie Mont Blanc, CNRS, IRD, IFSTTAR, ISTerre, 38000 Grenoble, France; 3 University Grenoble Alpes, CEA, CNRS, IRIG-SyMMES, STEP, 38000 Grenoble, France; 4 University Grenoble Alpes, CNRS, IRD, Météo-France, INRAE, Observatoire des Sciences de l’Univers de Grenoble (OSUG), UAR 832 CNRS, 38400 Saint Martin d’Hères, France; 5 Synchrotron SOLEIL, L’Orme des Merisiers, 91190 Saint Aubin, France; 6 University Grenoble Alpes, CEA, CNRS, IRIG-MEM, LEMMA, 38000 Grenoble, France

**Keywords:** quantum dot, indium, safer-by-design, environmental degradation, toxicity, EXAFS

## Abstract

Quantum dots (QDs) are widely used in optoelectronics, lighting, and photovoltaics leading to their potential release into the environment. The most promising alternative to the highly toxic cadmium selenide (CdSe) QDs are indium phosphide (InP) QDs, which show reduced toxicity and comparable optical and electronic properties. QD degradation leads to the release of toxic metal ions into the environment. Coating the QD core with robust shell(s) composed of another semi-conductor material enhances their properties and protects the QD from degradation. We recently developed double-shelled InP QDs, which proved to be less toxic than single-shell QDs. In the present study, we confirm their reduced cytotoxicity, with an LC50 at 77 nM for pristine gradient shell QDs and >100 nM for pristine thin and thick shell QDs. We also confirm that these three QDs, when exposed to simulated sunlight, show greater cytotoxicity compared to pristine ones, with LC50 ranging from 15 to 23 nM. Using a combination of spectroscopic and microscopic techniques, we characterize the degradation kinetics and transformation products of single- and double-shell QDs, when exposed to solar light at high temperature, simulating environmental conditions. Non-toxic pristine QDs degrade to form toxic In–phosphate, In–carboxylate, Zn–phosphate, and oxidized Se, all of which precipitate as heterogeneous deposits. Comparison of their degradation kinetics highlights that the QDs bearing the thickest ZnS outer shell are, as expected, the most resistant to photodegradation among the three tested QDs, as gradient shell, thin shell, and thick shell QDs lose their optical properties in less than 15 min, 60 min, and more than 90 min, respectively. They exhibit the highest photoluminescence efficiency, i.e., the best functionality, with a photoluminescence quantum yield in aqueous solution of 24%, as compared to 18% for the gradient shell and thin shell QDs. Therefore, they can be considered as safer-by-design QDs.

## 1. Introduction

QDs are semiconductor nanocrystals with remarkable optical properties such as bright photoluminescence that can be tuned with respect to the QD size and that shows low photobleaching. Currently, the main fields of application of QDs are optoelectronics, photovoltaics, lighting, and as biosensors [1]. For example, they are used as photoluminescent emitters in commercial TV screens and displays, and the development of QD-based light-emitting diodes (LEDs) is currently underway [2]. They are also expected to be used in the near future for photocatalysis purposes and they have been successfully developed, for instance, for polymerization purposes or for biomass valorization [3,4]. These uses, combined with inappropriate recycling, would lead to the release of potentially toxic QDs and/or their degradation products into the environment.

There is currently a great effort to develop safer and more sustainable nanomaterials, which would reduce environmental, health and safety (EHS) concerns, all along the life cycle of the product, while preserving its functionality [5,6]. Up to now, the most commonly used QDs have been Cd-based (e.g., CdSe, CdTe), but these are gradually being replaced by less toxic and RoHS-compliant InP QDs [7]. Although less toxic than CdSe [8], bulk InP is classified as probably carcinogenic by the International Agency for Research on Cancer [9]. Moreover, when addressing the toxicity of QDs, one should not only consider pristine QDs, but also the transformation products of QDs throughout their life cycle, especially after having been exposed to environmental conditions mimicking the end-of-life of QD-containing products.

The InP QDs presently on the market are core–shell structures in which the core semiconductor (InP) is passivated by a shell composed of another semiconductor material, typically ZnSe and/or ZnS, which protects the shell from quenching, thus enhancing photoluminescence properties [10,11,12]. Doping the InP core with Zn can further enhance the photoluminescence [13], as well as using a Zn(Se,S) gradient shell rather than a ZnS shell, because ZnSe shows a lower lattice mismatch with InP than Zn, which limits the formation of defect states at the core–shell interface [10]. Both approaches are considered as means to increase the performance of QDs, but they can also be regarded as safer and sustainable-by-design approaches. Indeed, reducing the lattice mismatch between the core and the shell ensures better protection of the core. Moreover, alloying the InP QD core with Zn reduces the overall content of potentially toxic elements, i.e., In, via substituting it with a less toxic element, i.e., Zn. Finally, their higher photoluminescence makes it possible to decrease the amount of QDs needed to achieve the same photoluminescence level in the final product.

Like Cd-based QDs, InP QDs are susceptible to photodegradation in environmental conditions, which reduces their photoluminescence and leads to the release of toxic chemicals. After photodegradation, we previously showed that QDs composed of an InZnP [13] or InZnSP [14] core (In:Zn, 1:1) capped with a Zn(Se,S) shell show increased toxicity as compared to the corresponding pristine QDs, due to the degradation of the QD structure [15]. When photodegraded, these QDs form large precipitates composed of In–carboxylate and –phosphate, and Zn–phosphate [15]. They would be better protected if a thicker shell was grown on their core, further enhancing their photostability. However, increasing the thickness of a Zn(Se,S) shell would imply increasing the Se content of the QD, which is not desired because Se is toxic at high concentrations [16]. Similar as other groups working on InP Qds (for review, see [10]), we recently reported the synthesis of InZnP QDs having a reduced In content in their core (InZnP, with an In:Zn ratio of 1:2 rather than 1:1 [15]) and being coated with three different shell types. These shells are either a single Zn(Se,S) gradient shell, or a double-shell where the Zn(Se,S) shell is further coated with a ZnS shell, either thin or thick [17]. The toxicity of pristine double-shelled InP QDs is lower than that of the corresponding single-shelled ones, but these QDs show substantial toxicity after aging in environmental conditions [18]. 

The objective of this study was to analyze whether these double-shell QDs are more resistant to photodegradation than single-shell QDs, which would ensure that their non-toxic, pristine state is maintained for longer times when exposed to environmental conditions. We assessed their photodegradation kinetics and characterized their degradation products after exposure to environmental conditions, in the aqueous phase. Upon irradiation with UV light, these QDs showed several successive stages of physico-chemical transformation. At each of these stages, the secondary products were characterized using scanning transmission electron microscopy and energy-dispersive spectroscopy, as well as X-ray absorption spectroscopy, which provided the speciation of indium, zinc, and selenium in the QD transformation products throughout the aging process.

## 2. Materials and Methods

### 2.1. Chemicals

Unless specifically indicated, all chemicals and reagents were purchased from Sigma Aldrich (Saint-Quentin Fallavier, France) and were >99% pure.

### 2.2. QD Synthesis and Transfer to the Aqueous Phase

**Figure 1:**
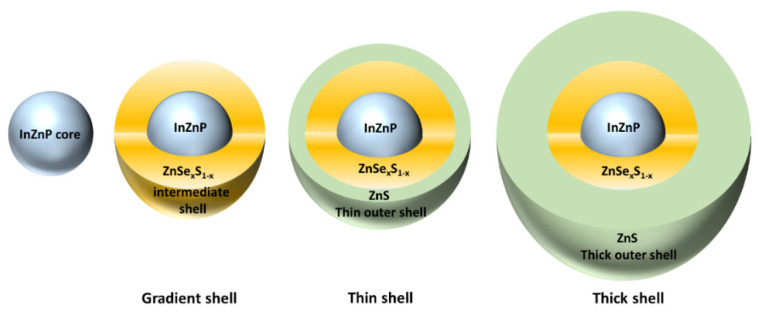
Schematic representation of the “gradient shell”, “thin shell”, and “thick shell” QDs.

QDs were synthesized as described previously [17]. Briefly, the InZnP core was synthesized as follows. Indium–myristate was prepared by mixing 6.9 mmol of indium acetate, 21.4 mmol of myristic acid, and 15 mL of octadecene (ODE) in a three-neck flask. The mixture was stirred, then degassed under vacuum for 3 h at 120 °C, then cooled at room temperature. The precipitate was washed with dry hexane (~150 mL), then dried under vacuum. Zinc oleate was prepared by mixing 5 mmol of zinc acetate, 10 mmol of oleic acid, and 9.35 mL ODE in a three-neck flask. After stirring and 1 h of degassing under vacuum at 120 °C, the solution was cooled down to room temperature and the flask was backfilled with Ar. This solution was stored in a glovebox until use. To prepare 0.4 mM TOP-Se and 0.4 M TOP-S stock solutions, we dissolved 2 mmol of selenium or 2 mmol of elemental sulfur in 5 mL trioctylphosphine (TOP). The solutions were stirred for 24 h. The core of InZnP nanocrystals was prepared by mixing 0.1 mmol of In–myristate with 0.2 mmol of Zn–stearate (ZnSt_2_) and 8.5 mL of ODE in a three-neck flask. The mixture was degassed for 1 h. Then, the flask was backfilled with Ar. The solution was heated to 300 °C in a molten salt bath and when the temperature reached 100 °C, we swiftly injected 0.1 mmol of (TMS)_3_P prepared in 1 mL of ODE. The reaction took place for 20 min, then we let the mixture cool down to 220 °C. A volume of 2.5 mL of the 0.4 M Zn–oleate solution was added dropwise, and then we added a mixture composed of 0.444 mL TOP-Se, 0.5 mL ODE, and 1.57 mL TOP-S stock solutions swiftly. This mixture was heated to 300 °C with a heating ramp of 10 °C per min. After 30 min of gradient shell growth, the reaction was stopped by cooling down to room temperature. This resulted in the QD called “gradient shell”, which was further washed three times by precipitation using methanol/chloroform and acetone (1:1, *v*/*v*), and redispersed in chloroform. They were stored in hexane. For double-shell QDs preparation, the gradient shell QDs were not cooled to room temperature after synthesis, but rather kept at 230 °C. Zinc ethylxanthane (0.1 mmol) was dissolved in 100 µL of DMF and 1 mL of toluene, then mixed with 0.8 mmol of ZnSt_2_ dissolved in 3 mL of ODE. At this step, the Zn–ethylxanthane mixture was injected in the ZnSt_2_ solution using a syringe pump so that the injection rate could be strictly controlled. The latter was fixed at 8 mL/h. This reaction was maintained for 30 min or 60 min, to grow a thin or a thick ZnS shell, respectively, on the gradient shell QD, leading to the QDs being called “thin shell” and “thick shell”, respectively. Then the reaction was quenched by lowering the temperature to room temperature. These three types of QDs are represented schematically in Figure 1.

**Figure 2:**
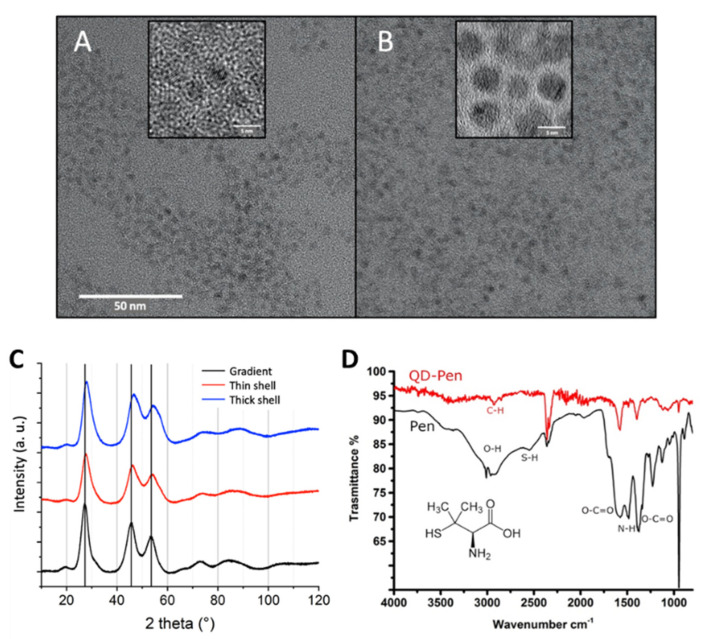
Characterization of the QDs. Cryo-TEM images of gradient shell QDs (**A**) and thick shell QDs (**B**) at the same magnification (insets: higher magnification images). (**C**) X-ray diffraction patterns of the three QD samples. (**D**) FTIR spectrum of penicillamine-capped gradient shell QD (red); the same spectrum is obtained for the different types of shells.

The QDs were synthesized, then transferred to the aqueous phase using ligand exchange with D-penicillamine, as previously described [17]. For this purpose, a solution of 0.2 M D-penicillamine was prepared in MilliQ water and degassed for 15 min with Ar. We then added 200 µL of Tris (2-carboxyethyl) phosphine hydrochloride (TCEP) to the reaction to avoid disulfide bond formation, and adjusted the pH to 9 with a 25 wt% solution of tetramethylammonium hydroxide (TMAOH) prepared in methanol. The solution was degassed for 15 min with Ar. Then, 0.5 mL of this D-penicillamine solution was added to 1 mL of QD suspension at 3–5 µM, also previously degassed. This mixture was biphasic. It was stirred for 45 min at room temperature, then centrifuged to separate the two phases. The upper phase, which contained the QDs, was sampled and purified using a NAP10 size exclusion column (Sephadex G-25 DNA grade, GFischer Scientific, Illkirch, France). This removed excess ligands and remaining traces of chemical products from the synthesis. The columns were calibrated either with phosphate-buffered saline (1X PBS) buffer or with deionized water; the same were used as eluent and storage mediums for the QDs. They were kept at 4 °C in the dark and under an argon atmosphere, in order to reduce as much exposure as possible to oxygen from the air and to UV light. In these conditions, they remained stable for 1 year when transferred in PBS and for more than 3 years when transferred in ultrapure water, without any change in their optical properties or any sign of precipitation. Their physico-chemical characteristics (primary size, composition, size distribution, nr. of shells, zeta potential, photophysical properties) are summarized in Table S1 and cryo-transmission electron microscopy (cryo-TEM) images, X-ray diffraction patterns and FT-IR analyses are shown in Figure 2. As expected, thick shell QDs are larger than gradient shell QDs (Figure 2A,B), and the diffraction peaks shift to larger angles with increasing ZnS shell thickness (Figure 2C) due to the higher compressive strain, leading to a lower lattice parameter of the heterostructure. Moreover, the FTIR spectrum shows that, in comparison with free penicillamine (Pen, black), the sulfhydryl signal at around 2550 cm^−1^ is absent after coordination to the QD surface (Figure 2D). The COO^−^ asymmetric stretching band remains predominant at 1600 cm^−1^, while the NH^+^ bending signal at 1500 cm^−1^ becomes much lower in bound pen. The COO^−^ symmetric stretch exhibits a slight shift from 1400 to 1410 cm^−1^. (N.B.: The bands at around 2350 cm^−1^ correspond to CO_2_ from the working atmosphere.)

More characterizations of these QDs (elemental composition based on EDX analysis, XPS spectra) are available in our previously published article describing their synthesis with more details [17].

### 2.3. Accelerated Weathering in a Climatic Chamber

The QDs were weathered in a Q-SUN Xe-1 Xenon arc test chamber (Q-lab, Bolton, UK) that provides a full spectrum sunlight. An irradiance of 1.4 W/cm² and a temperature of 40 °C were used for these weathering, following the ISO norms 4892-1 (2000) and 4892-2 (2013) and corresponding to the weather conditions of a sunny day at noon in the equator region. QDs were diluted to 1 µM in PBS or MilliQ water (2 mL total volume), and weathered in 10 mm × 10 mm quartz cuvettes that were closed with a screw cap. All QDs were aged for 24 h in these conditions with direct measurement and sample collection at different aging times, for further characterization.

### 2.4. Characterization of QD Properties

QD size was measured on cryogenic-TEM images, obtained with a FEI (Eindhoven, Netherlands) Polara microscope operating at 300 kV and images were recorded on a Gatan (Elancourt, France) K2 camera. Samples were prepared by depositing 4 µL of diluted QDs onto a 400-mesh copper grid coated with a homemade carbon film. Size distribution was measured as Z-average and polydispersity index (PdI) via dynamic light scattering on a nanoZS zetasizer (Malvern, Palaiseau, France). Their elemental composition was analyzed by energy-dispersive X-ray analysis (EDX) using an Ultra 55+ scanning electron microscope (Zeiss, Marly le Roi, France) equipped with an EDX probe. For this analysis, a concentrated drop of QDs was deposited on a clean silicon substrate and allowed to dry at room temperature. Photoluminescence quantum yield was measured using an integration sphere (Hamamatsu, Massy, France, Quantaurus Absolute PL quantum yield spectrometer) at room temperature, on the QD colloidal solution. The UV-visible absorbance (UV-vis abs) was measured using an 8452A spectrometer (Hewlett Packard, Puteaux, France) and the steady-state photoluminescence (PL) was measured using a Fluorolog FL3-22 spectrometer (Horiba-Jobin Yvon, Palaiseau, France) equipped with a 450 W xenon lamp and a double-grating monochromator for excitation and emission, as well as a photomultiplier tube (R928 Hamamatsu, Massy, France) for detection. Absorption and emission spectra were measured at 0 min, 15 min, 30 min, 45 min, 1 h, 1 h 30, 2 h, 4 h, 6 h, 24 h of aging. Their crystal structure was determined using a Panalytical X’Pert diffractometer equipped with a copper anode (λKα1 = 1.5406 A and λKα2 = 1.5444 A) and including a X’Celerator 1D detector, and FT-IR spectra were obtained using a Bruker (Wissembourg, France) ALPHA E, 200,396 spectrometer.

### 2.5. Cell Culture and Cytotoxicity Assay

Human skins were obtained from breast surgery of healthy female donors with their informed consent (Centre Hospitalier Universitaire de Grenoble, France), and following the French Public Health Code, article L1245-2 on the use of surgical waste for research purpose. The samples were treated anonymously, and their collection and storage were declared according to the French law, under the CODECOH DC-2008-444 document. Keratinocytes were extracted from these skins using a previously reported procedure [19] and then grown in Keratinocyte serum-free medium (KSF-M) supplemented with 25 μg/mL bovine pituitary extract, 1.5 ng/mL epidermal growth factor (EGF) and 75 μg/mL primocin. They were maintained at 37 °C and 5% CO_2_ in a humidified atmosphere. These cells were used between passage 1 and 3. For cytotoxicity evaluation, cells were seeded in transparent 96-well plates at 25,000 cells per well and exposed 24 h later to QD suspensions at concentrations ranging between 3.125 and 100 nM. Cells exposed to KSF-M were only used as a control, and cells exposed to Triton 1% were used as a positive control. After 24 h of exposure, 50 µL of exposure medium was sampled from each well and transferred to a clean 96-well plate. Lactate dehydrogenase (LDH) release was quantified using In Vitro Toxicology Assay Kit, Lactic Dehydrogenase based (Merck Sigma-Aldrich, St Quentin Fallavier, France) following the manufacturer’s instructions. The cytotoxicity experiment was repeated three times on keratinocytes from three distinct donors (*n* = 3). Each of these experiments included five technical replicates per concentration. Statistical analysis was performed using R-studio, first via the Kruskal-Wallis test, then using pairwise comparison via the Wilcoxon rank sum test. A result was considered statistically significant (*) when *p* < 0.05.

### 2.6. Scanning Transmission Electron Microscopy Coupled to Energy-Dispersive X-ray Analysis

Pristine and aged QDs were imaged using scanning transmission electron microscopy (STEM) with a FEI/Tecnai OSIRIS microscope (Eindhoven, The Netherlands) operated at 200 kV. A droplet of QDs was deposited on a copper grid coated with a carbon film, then allowed to dry for 24 h at room temperature. Samples were stored in the dark until imaging. Imaging was performed in the high angle annular dark field (HAADF) mode, and the selected area was analyzed using energy-dispersive X-ray analysis (EDX). 

### 2.7. X-ray Absorption Spectroscopy

After 30 min, 2 h, 6 h and 24 h of aging, significant changes of the QDs’ physico-chemical properties were observed, such as aggregation and sedimentation or color modification. Therefore, aged QDs were collected at these time-points for further analysis by X-ray absorption spectroscopy (XAS). When aggregation was observed macroscopically, samples were centrifuged at 1000× rcf for 2 min and the pellet and supernatant were sampled separately and mixed with 20% glycerol. These samples were placed in the dedicated sample holder and immediately immersed in liquid nitrogen. They were stored in liquid nitrogen until analysis. Zinc speciation was analyzed on the FAME (BM30B) beamline at the European synchrotron radiation facility (ESRF, Grenoble, France) [20] using the previously reported protocol and settings [15]. For these analyses, the beamline was operated in a multi-bunch mode (150 to 200 mA). Indium and selenium speciation were similarly analyzed at In K-edge and Se K-edge on the SAMBA beamline at the SOLEIL synchrotron (Optimized Light Source of Intermediate Energy to LURE, Saclay, France) [21] operating at 450 mA in top-up mode. The SAMBA beamline is equipped with a Si(220) monochromator running in a continuous scan acquisition mode, while FAME beamline is equipped with a 35-element Ge detector (Canberra, Montigny-le-Bretonneux, France). On both FAME and SAMBA beamlines, the samples were analyzed in a liquid He cryostat, i.e., at 20 K. Ten to forty spectra of 3 min each were averaged, depending on the signal. For Se, we observed photoreduction of the samples aged for 24 h due to the beam irradiation, therefore only the five first spectra were averaged for further signal analysis. In all other samples, no radiation damage was observed. Spectra analysis was performed as follows. First, the spectra recorded on each sample were calibrated by setting the absorption edge at 12,658.0 and 27,940.0 eV, thanks to the use of spectra recorded simultaneously on reference Se and In foils. Then, these spectra were normalized using the Demeter software package, version 9.26 [22]. Finally, shell fitting was used to analyze the spectra from pristine QDs and linear combination fitting (LCF) for aged QDs analysis, using the Demeter/ARTEMIS and ATHENA softwares version 9.26, respectively [22]. For LCF, we used the following reference spectra: pristine QDs, In acetate and In phosphate (In K-edge), ZnS (bulk and nano), hopeite (Zn_3_(PO_4_)_2_, 4H_2_O), Zn phosphate dehydrate (Zn_3_(PO_4_)_2_, 2H_2_O) and Zn-reacted hydroxylapatite at pH 6 as previously described [15], Zn–GSH (1:10 mol:mol, pH 4.5), and Zn–malate as a proxy for Zn–carboxylate (Zn-COOH) (Zn K-edge). Reference spectra used for selenium speciation were red selenium (red Se(0)), grey selenium (grey Se(0)), sodium selenite (Na_2_SeO_3_), sodium selenate (Na_2_SeO_4_), selenium dioxide (SeO_2_), zinc selenide (ZnSe), selenium disulfide(SeS_2_) selenocystine, selenomethionine, and selenourea. Spectra for the aged QDs were analyzed by LCF using pristine, red Se(0) and Na_2_SeO_3_ reference spectra.

## 3. Results

### 3.1. Photophysical Characterization of Pristine and Aged QDs

Pristine “gradient shell”, “thin shell”, and “thick shell” QDs were characterized after ligand exchange using penicillamine as a surface ligand and purification. Their characteristics are summarized in Table S1 and Figure S1. The three types of QDs showed an excitonic peak at 518 nm (gradient shell and thick shell QDs) or 512 nm (thin shell QD) when analyzed via UV-vis spectroscopy (Figure S1A), and a maximum photoluminescence (PL) emission at 565–571 nm when analyzed via fluorescence spectroscopy (Figure S1B). The corresponding core-only QD showed an excitonic peak at 475 nm and a maximum PL emission at 530 nm (Figure S1C). This is typical of indium phosphide core/shell structures with zinc sulfide or selenide, the large band offsets, and type I band alignment, leading to a confinement of electrons and holes in the core. Therefore, only a comparably small redshift (typically 40–50 nm) is observed when adding the first intermediate shell (gradient Zn(Se,S)). As expected, no further shift occurred during the subsequent deposition of the outer ZnS shell. For comparison, in CdSe/CdS core/shell structure, the quasi-type II band alignment leads to the delocalization of the electron wavefunction into the CdS shell, inducing a pronounced redshift of the absorption and PL emission spectra.

The relative PL intensities of the gradient shell and thin shell QDs were lower than that of the thick shell QDs, with PL quantum yields in the aqueous phase of around 18% for the gradient and thin shell QDs and 24% for the thick shell QDs, respectively (Table S1). These values are, as widely observed upon the aqueous phase transfer via the surface ligand exchange, significantly smaller compared to those measured in the organic phase where QDs are produced (55–70%) [17].

**Figure 3:**
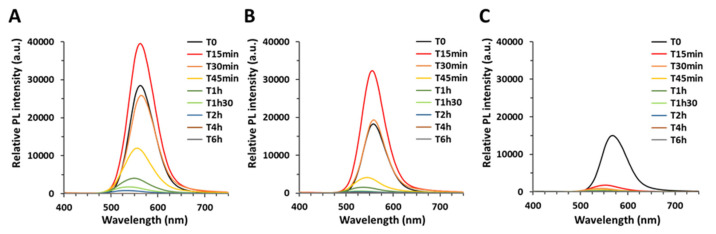
Monitoring of the QDs’ photoluminescence during the aging process. Corrected PL spectra of thick shell (**A**), thin shell (**B**), and gradient shell (**C**) QDs. For each set of spectra, the data were corrected to reach identical absorbance at the excitation wavelength of 400 nm.

Artificial weathering in environmental conditions caused progressive loss of the PL emission (Figure 3) and of the characteristic excitonic peak at 512 or 518 nm (Figure S2), which proves QD degradation. For both the thin shell and thick shell QDs (Figure 3A and Figure 3B, respectively), the PL first increased after 15 min of weathering, which corresponds to the elimination/photo-curing of surface defects [23]. Then the PL of both types of QDs decreased progressively with weathering duration, finally reaching <10% of the initial PL after 1 h and 1 h 30 of weathering of the thin shell and thick shell QD, respectively (Figure 3A,B).

In contrast, gradient shell QDs degraded very rapidly (Figure 3C). The initial PL increase was not observed, certainly because it occurred before 15 min of aging, and ~10% of the initial PL was reached after only 15 min of aging of these QDs.

These results were confirmed using UV-vis spectroscopy, i.e., initially, the three QDs were intact, with a well-defined excitonic peak (Figure S2A). After only 15 min of weathering, the excitonic peak was no longer observed for the gradient shell QDs, in contrast to the thin and thick shell QDs (Figure S2B). The excitonic peaks of thin and thick shell QDs were less intense after 30 min of weathering (Figure S2C); they totally disappeared after 45 min (Figure S2D). Moreover, during the weathering experiment, the three QDs precipitated and their color progressively changed, as illustrated in the insets of Figure S2. While pristine QDs formed a homogeneous bright orange suspension (Figure S2A, inset), after 15 min of weathering the QDs settled down as an orange precipitate (Figure S2B, inset), which then lightened and became yellow after 30 min (Figure S2C, inset). It was pink (Figure S2D, inset) and grey after 45 min and 2 h, respectively, and then it progressively lost its color (Figure S3A, recorded after 24 h of aging).

This behavior confirms the greater photostability, i.e., better resistance to photodegradation in environmental conditions, of double-shell QDs as compared to the single, gradient shell QDs. When the QDs were only exposed to a higher temperature (37 °C) without irradiation by simulated sunlight, such a sequential degradation was not observed (Figure S3B), proving that the observed behavior is due to a photo-degradation process.

### 3.2. Toxicity of Pristine and Aged QDs

**Figure 4:**
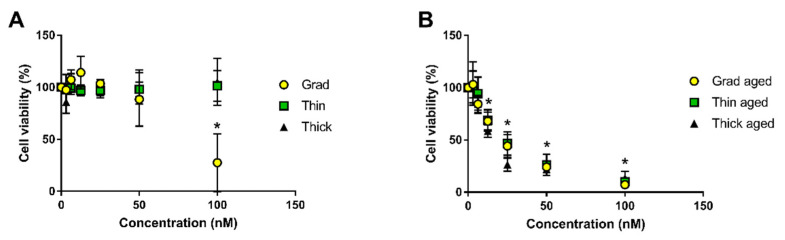
Cytotoxicity of pristine and aged QDs towards human primary keratinocytes. Pristine QDs (**A**) and aged QDs (**B**). Cell viability was evaluated via measurement of LDH release, representative of cell membrane damage. Statistical analysis, * *p* < 0.05, exposed vs. control (medium), *n* = 3.

We previously reported that all three aged QDs drastically reduced cell viability while only the gradient shell pristine QD altered it, via measurement of cell metabolic activity using WST-1 assay [18]. In the present study, this was confirmed via measuring the impact of pristine and aged QDs on cell membrane integrity, using the LDH assay. For this experiment, QDs were weathered for 24 h in order to reach total degradation of the three compositions. Among pristine QDs, we confirmed that only gradient shell QDs reduced cell viability, and only at the highest tested concentration, which was 100 nM (Figure 4A). Conversely, the three aged QDs were cytotoxic to human primary keratinocytes, with a significant reduction of cell viability from 12.5 nM (Figure 4B). No significant difference of toxicity was observed between the three aged QDs, except at 25 nM where aged thick shell QDs were more cytotoxic than gradient shell and thin shell QDs. Analysis of these cytotoxicity curves permitted the determination of the lethal concentration 50 (LC50), i.e., the concentration leading to 50% of cell death. It was 77 nM for pristine gradient shell QD, 22 nM for aged gradient shell QD, 23 nM for aged thin shell QD, and 16 nM for aged thick shell QD.

### 3.3. Physico-Chemical Characterization of QDs during the Aging Process

As weathering drastically increased the cytotoxicity due to QD degradation, we characterized the physico-chemical transformation encountered by the QDs during the aging process and we identified their transformation products.

#### 3.3.1. STEM-EDX Analysis

**Figure 5:**
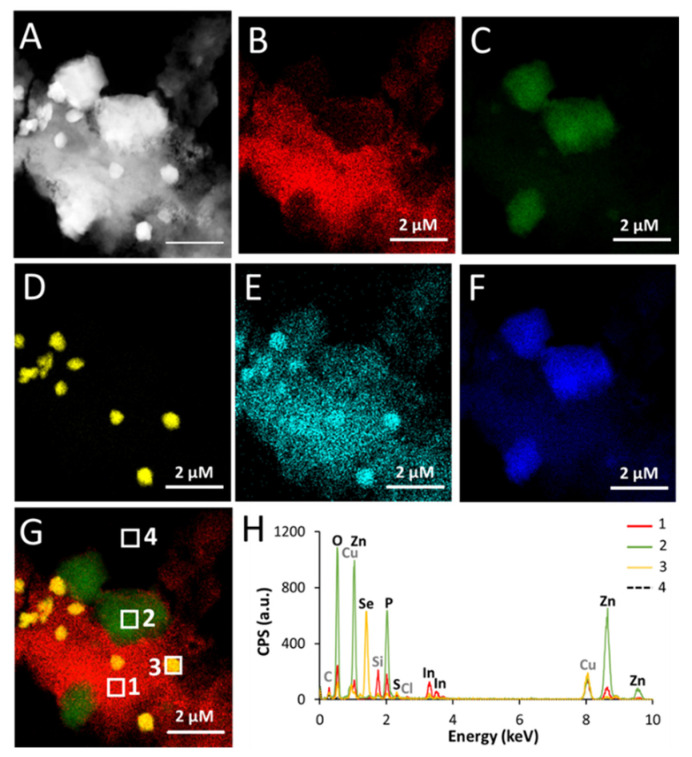
Characterization of aged thick shell QDs by STEM-EDX. (**A**) STEM (HAADF) imaging of aged thick shell QD, (**B**–**F**) elemental distributions of In (**B**), Zn (**C**), Se (**D**), S (**E**), and P (**F**). (**G**) merge of (**B**,**C**,**D**), showing four regions from which EDX spectra were extracted (regions indicated as 1, 2, 3, 4 in G); these DX spectra are reported in (**H**).

The diameter of pristine QDs ranged from 3.7 nm (gradient shell), 4.8 nm (thin shell) to 6.1 nm (thick shell) ([17] and Table S1). TEM images showed isolated, non-aggregated nanocrystals (Figure 2A,B). In contrast, after 24 h of weathering in PBS, all three types of QDs appeared as large precipitates when observed by STEM (Figure 5A, Figures S4A and S5A for thick shell, gradient shell, and thin shell QD, respectively). Heterogeneous aggregation was observed regardless of the QDs, where indium (In) co-precipitated with some zinc (Zn), sulphur (S), and phosphorus (P) (Figure 5B–G, Figures S4B–G and S5B–G). EDX analysis of regions 1 from Figure 5G, which is representative of these indium-rich regions of the precipitates from thick shell QDs, showed that indium also co-precipitated with carbon (C) and oxygen (O) (Figure 5H). The same was observed when analyzing gradient shell and thin shell QDs (Figures S4 and S5). Most of the Zn co-precipitated in distinct regions with some P (Figure 4F and Figure 5C), for example region 2 in Figure 5G is representative of these Zn-rich areas (the same is true for gradient shell and thin shell QDs, see Figures S4 and S5). EDX analyses showed that the Zn-rich regions also contained some O (Figure 5H, Figures S4H and S5H). Finally, Se co-precipitated with S in distinct regions (Figure 5D,E for thick shell QDs and Figures S4E,F and S5E,F for gradient shell and thin shell QDs).

When QDs were aged in pure water instead of PBS, heterogenous aggregation was also observed (Figure S6A), but no segregation of In and Zn was observed. In and Zn co-precipitated in regions also containing C, O, and P (Figure S6B–D). In this condition, Se also co-precipitated with S separately from In and Zn (Figure S6E,F).

These results demonstrate that artificial weathering in environmental conditions led QDs to dissolve and precipitate.

XAS analyses were performed to identify more precisely the transformation products occurring after QD weathering.

#### 3.3.2. XAS Analysis

The chemical speciation of In, Zn, and Se was characterized in both pristine and aged QDs by X-ray absorption spectroscopy (XAS, including X-ray Absorption Near Edge Structure (XANES) and extended X Ray absorption fine structure (EXAFS)). Both XANES and EXAFS are sensitive to the local order around the target element selected by its absorption edge [24]. XANES is directly sensitive to the oxidation state of the analyzed element. EXAFS allows a precise determination of the distance, number, and nature of the neighboring atoms around the analyzed element [24].

First, we analyzed indium speciation in pristine and aged QDs (Table 1). In pristine QDs, indium was coordinated with 4.0–4.2 P atoms at a bond distance of 2.53 Å, typical of crystalline InP in the zinc blende structure [25]. A minor contribution of Se atoms at 2.55 to 2.57 Å, characteristic of amorphous InSe [26], enhanced the quality of the fit. The EXAFS spectra used for this analysis and their respective Fourier-transformed spectra are shown in Figures S7 and S8, with contributions of P and Se atoms illustrated in Figure S7B. Similar conclusions were obtained for the three pristine QDs. In the pristine gradient shell QD sample, a slightly longer InSe bond length was found, combined with a smaller σ² value (Table 1), which suggests that the InSe clusters are better crystallized. This can be interpreted as the presence of a mixed layer at the core–shell boundary or as the consequence of InZnSeP alloy formation [27]. Considering the occurrence of both S and Se in the QD composition (S in the core, Se and S in the gradient shell), both elements could contribute to the coordination sphere of In in pristine QDs. When fitting the spectra with S instead of Se, the results were of equivalent quality for pristine thick shell QDs. However, the fitting quality was better when using with Se instead of S for pristine thin shell and gradient shell QDs. This result demonstrates that the interface of the gradient shell is composed of both ZnS and ZnSe. In summary, in pristine QDs, In was coordinated with P in the typical InP structure, with some contribution of Se and S signing their presence at the QD core–shell boundary.

**Figure 6:**
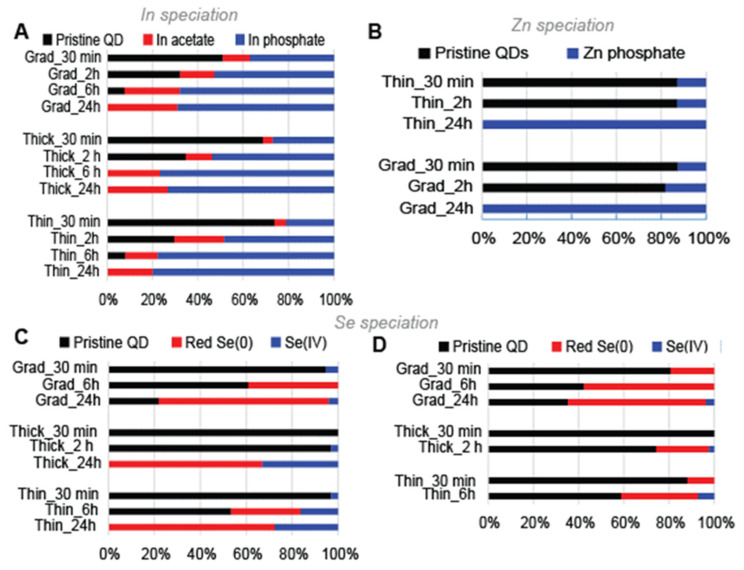
Proportion of In, Zn, and Se species forming upon QD irradiation. These proportions are derived from linear combination fit of EXAFS spectra at the In K-edge (**A**), at the Zn K-edge (**B**), and from XANES and EXAFS spectra at the Se K-edge ((**C**,**D**), respectively).

Throughout QD aging in simulated environmental conditions, drastic changes of the In speciation were observed. The +III redox state remained unchanged as proven by the maximum of the absorption edge in the XANES spectra being at 27.95 keV in both pristine and aged QDs (Figure S9). A gradual loss of the typical In–P coordination was observed, characterized by the peak at 2 Å in Fourier-transformed EXAFS spectra (Figures S7D and S8B). It was progressively replaced by an In–O coordination, with the emergence of a characteristic peak at 1.65 Å in the Fourier-transformed spectra (Figures S7D and S8B), similar to the position of the main peak in In–phosphate and In–acetate reference compounds (Figure S7D). After 30 min of aging, the intensity of this In–O contribution was higher in the aged gradient shell QD sample (Figure S7D), compared to aged thin and thick shell QDs (Figure S8B), suggesting that the gradient shell QDs were more degraded than the other QDs. After 24 h of aging, all three QD samples showed only this 1.65–1.7 Å contribution and no more than the 2 Å peak, proving complete degradation (Figures S7D and S8B). To determine which species could be responsible for the observed In–O coordination, we used linear combination fitting (LCF) of EXAFS spectra. In–phosphate and In–acetate (used as a proxy for In–carboxylate) reference spectra were used in LCFs. This analysis showed the coexistence of In–carboxylate and In–phosphate in all aged samples (Figure 6A, the numerical values are reported in Table S2), with the ratio of In–phosphate to In–carboxylate progressively increasing with aging duration. Therefore, aging destroyed the InP crystal phase in the QD cores and led to their transformation to In–phosphate and In–carboxylate.

Then, we analyzed Zn speciation in pristine QDs. The thick shell QDs were not analyzed owing to their similar structure as that of thin shell QDs. The oxidation state of Zn did not change during the aging experiment, as visible by the unchanged XANES spectra (not shown). In both the gradient shell and thin shell pristine QDs, the first coordination sphere around the Zn atom was composed of S and Se atoms in a tetrahedral coordination, at a distance of 2.34 and 2.45 Å, respectively (Table 1, and EXAFS spectra and Fourier-transformed EXAFS spectra are shown in Figure S8). These values are typical of ZnS and ZnSe in the zinc blende structure [29,30]. Higher coordination spheres were dominated by the presence of Zn, with a much lower number of atoms than in the ZnS and ZnSe crystalline phases (Table 1), suggesting a structural disorder that can be explained as follows. First, the presence of a mixed ZnSe_x_S_(1−x)_ phase in the gradient shell, whose structure is likely disordered, could introduce such variations. Second, the presence of phases with various S/Se ratios within the shell can lead to additional destructive interferences. Finally, for the thin shell QD, the additional presence of ZnS in the outer shell further introduces disorder in the signal. As expected, the thin shell QDs that contain this additional ZnS shell, show a higher coordination number for S atoms (3.0 S and 1.4 Se, compared to 2.6 S and 1.5 Se, respectively, Table 1).

The speciation of Zn also evolved during the aging process. Both gradient shell and thin shell QDs showed similar behaviors, with no change in the oxidation state (XANES region of the spectra), but formation of minor amounts of Zn phosphate (10 to 20% of total Zn) after 30 min and 2 h. ZnS and Zn(Se,S) from the shells were completely transformed into Zn phosphate after 24 h (Figure 6B and Table S2). Again, phosphate may originate from the PBS buffer, and possibly from the oxidation of InP. Therefore, as for In, aging led to destruction of the Zn(Se,S) crystal structure and to the formation of Zn–phosphate.

Lastly, we analyzed Se speciation in these QDs. The XANES regions of the spectra show that the oxidation state of Se was close to that of ZnSe in pristine QDs, with a position of the absorption edge at 12,662 eV (Figure S11A). Aging caused oxidation of Se to Se(0) and selenite (Se(IV)) to a minor extent, with the absorption edges shifting to 12,660 eV and 12,663 eV, respectively (Figure S11A). In samples aged for 24 h, the gradient shell QDs contained mostly Se(0), together with a minor fraction of reduced Se, which represented 22% and 39% of the total Se, based on XANES and EXAFS LCFs, respectively (Figure 6C–D, respectively and Table S2). In the thin and thick shell QD samples, Se was totally oxidized and contained about 70% Se(0) and 30% Se(IV), based on XANES LCF (Figure 6C and Table S2). LCFs could not be analyzed from EXAFS data for thin shell and thick shell QDs aged for 24 h due to beam damage affecting the samples.

Finally, Se EXAFS spectra, their Fourier transformation, and their shell fits are shown in Figure S11B,C, respectively. The coordination sphere around the Se atoms in pristine QDs, deduced from EXAFS analysis, was dominated by a first shell composed of four Zn atoms at 2.46 Å (Table 1), similar as in bulk ZnSe crystallizing in the zinc blende structure [30]. Contributions of atoms from higher electron shells were very weak compared to crystalline ZnSe, which is consistent with the presence of a disordered ZnSe_x_S_(1−x)_ phase.

## 4. Discussion

In the present article, we analyze the physico-chemical transformation of three different InP QDs when exposed to artificial weathering under simulated sunlight, which leads to a significant increase in their cytotoxicity.

Our main conclusions are that when exposed to simulated sunlight, QDs lose their absorption and photoluminescence as they degrade. Gradient shell QDs degrade more rapidly than thin shell QDs, themselves degrading more rapidly than thick shell QDs. This behavior confirms the greater photostability, i.e., better resistance to photodegradation in environmental conditions, of double-shell QDs as compared to the single, gradient shell QDs. Since pristine QDs show minor cytotoxicity while aged QDs are highly cytotoxic, avoiding QD degradation is an efficient measure to reduce their hazard. Our results also confirm that thick shell QDs show the highest initial brightness. Therefore, double-shelled QDs with a thick outer shell are good safer-by-design QD candidates as compared to the two other QDs, since their functionality, i.e., photoluminescence, is better and their potential toxicity would be reduced due to their lower photodegradability. During the weathering process, we first observe QDs settling down, while their fluorescence properties remain intact if they are re-dispersed by shaking the suspension. Our hypothesis is that it is certainly due to loss of surface ligands, as previously described for CdSe QDs [20].

Longer weathering times lead to the progressive chemical degradation of these three QDs, with the formation of secondary products showing distinct optical properties. Aging of CdSe QDs in acidic or alkaline conditions has been shown to induce degradation of the surface coating and release of Cd ions due to the oxidation of Se [20,21]. The InP QDs studied here also contain the oxidation-sensitive elements Se and S, therefore some redox reactions can be expected during weathering, and indeed EXAFS analysis proves that oxidation of Se occurs. This would potentially damage QD outer shell layers, i.e., weaken QD core protection, allowing ligands to reach the core, and subsequently lead to its gradual degradation.

Regarding the transformation products, EXAFS analysis shows that In and Zn released from QDs form complexes with phosphates and carboxylates. According to Pearson’s principle (HSAB principle), In^3+^ is a hard acid and Zn^2+^ is a borderline (intermediate) acid [31]. Therefore, these two elements preferentially complex with hard bases, including carboxylates and phosphates [31]. The precipitates observed in the EDX analyses of QDs aged in PBS suggest that Zn precipitates with phosphates (co-localization of Zn, P, and O in the same regions) and that In precipitates with carboxylates, phosphates, and thiols (co-localization of In, C, P, S, and O). A segregation of In and Zn is observed in these precipitates, which can be attributed to their different affinity for the complexing agents. Moreover, the stability constant of hoepite, which is the most frequently reported Zn–phosphate species, is 10^−35^ [22], while the stability constant of In–orthophosphate is 10^−25^ [23]. Such difference would lead to distinct precipitation kinetics, which can explain the observed segregation of In–phosphate and Zn–phosphate precipitates. In comparison, when QDs are aged in water, such segregation of In and Zn precipitation does not occur. It is probable that, in this condition, In and Zn ions form aquo or hydroxide species that co-precipitate simultaneously.

The source of phosphates and carboxylates that complex In and Zn ions when QDs degrade can be multiple. QDs are coated with penicillamine that bear one –COOH and one –SH moiety. Moreover, weathering is performed in aerobic conditions, i.e., in the presence of O_2_ and CO_2_ from ambient air, and CO_2_ can dissolve in the QD suspension and would be another source of carboxylates. Furthermore, QDs are weathered in phosphate buffer saline, which contains 10 mM of phosphate. The contribution of phosphate resulting from the oxidation of indium phosphide is also possible. However, the phosphate concentration in the PBS buffer (~10 mM) is around 50 times higher than the concentration of P in the QDs (~1 µM, around 200 P atoms per 2.7 nm InZnP core). This suggests that PBS is the most probable source of phosphate ligands. Therefore, our interpretation is that phosphates mainly originate from the PBS buffer in which the QDs are aged and carboxylates from penicillamine and/or CO^2^ originating from ambient air that might dissolve in the QD suspension.

**Figure 7:**
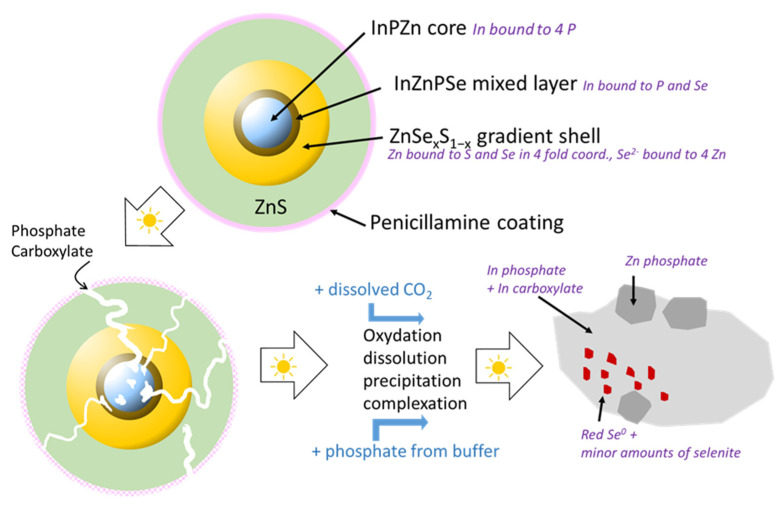
Scheme of the proposed processes taking place during aging of the QDs under simulated sunlight exposure. Photodegradation leads in a first stage to the loss of penicillamine surface ligands inducing the precipitation of the QDs. Partial degradation of the ZnS outer and Zn(Se,S) intermediate shells gives access for complexing agents, such as phosphate, to the InZnP, leaching In3+ in form of In-phosphate and -carboxylate. Finally, the core and the shell undergo complete dissolution, leading to oxidation products such as red Se(0), SeO_3_^2−^, SeO_4_^2−^, SO_4_^2−^ and PO_4_^3−^. The secondary phases result from the precipitation/complexation of the released metal cations with these anions present in solution. The kinetics of the different stages of photodegradation depend on the core/shell structure: they are fastest for the gradient shell system without additional outer shell, and slowest for the core/shell/shell structure with a thick ZnS shell.

Based on these results, a scenario of weathering in environmental conditions can be proposed (Figure 7). In pristine QDs, In is present as indium phosphide (InP) in the core, and some indium selenide (In_2_Se_3_) or a mixed In(P, Se) is present at the core–shell boundary. Furthermore, an amorphous layer of mixed oxides (InPO_3_, InPO_4_, In_2_O_3_, In(OH)_3_, InO(OH)) can be expected on the surface of the InZnP core according to Virieux and coworkers [32]. The latter is formed during the high temperature QD synthesis due to the in situ generation of water induced by the ketonization of fatty acids used for complexing the In and Zn precursors [32]. This layer is not detected in the EXAFS analysis because the contribution of these oxides must be lower than 10%, which is the limit of detection of a species with this technique [24]. Zn is bound to P in the InZnP QD core, to S and Se in the ZnSe_x_S_1−x_ phases of the gradient shell, and in the ZnS phase of the thin or thick outer shell, in the expected tetrahedral coordination in all phases. Se is only present in the gradient shell and bound exclusively to Zn. Upon exposure to environmental conditions, some dissolution, oxidation, complexation, and precipitation processes occur. 

Up to 30 min of weathering, the precipitation of all types of QDs indicates at least partial desorption of the penicillamine surface ligands inducing QD aggregation. Surprisingly, in this short lapse of time already between 30% (thin shell) and 50% (gradient shell) of In is present as In–carboxylate and phosphate, while only around 15% of Zn is transformed to Zn–phosphate and between 0% (thick shell) and 20% (gradient shell) of Se is oxidized. This result suggests that metal ions can leach from the core prior to the complete shell dissolution. Unfortunately, up to now, most QD stability studies focus on the leaching behavior of the core elements (e.g., Cd^2+^ in the case of CdSe/ZnS QDs) due to their higher toxicity, neglecting the evolution of the shell constituents. Nonetheless, a similar behavior has been recently reported by Paydary and Larese–Casanova [33], who studied the dissolution kinetics of aqueous solutions of CdSe/ZnS QDs in the dark. The simultaneous release of Zn^2+^ and Cd was attributed to non-uniform coverage of the CdSe cores by the ZnS shell. Here, considering that after 30 min of aging the thin and thick shell QDs still exhibit similar PL properties in terms of intensity, peak position, and line width as the pristine QDs, indicates that In–carboxylate and In–phosphate generated during this period stems from a small fraction of less well passivated and hence non-emissive QDs in the ensemble.

Only for longer irradiation times starting from 45 min, the decrease in PL intensity is accompanied by a blue shift, which is a clear sign of shrinkage of the emissive core. A completely different situation is observed in the gradient shell QDs, which show a strong decrease in PL intensity and a blue shift already after 15 min of irradiation. After 30 min, already around 50% of the In is transformed to In–carboxylate and In–phosphate, and around 20% of Se^2−^ is oxidized to Se(0). However, once again only a small amount of Zn (10–15%) is transformed to Zn–phosphate species, which shows that this parameter is not a reliable indicator of the shell dissolution. Several factors can explain the comparably fast degradation kinetics observed for the different QDs in this comparative study [32]: first, complexing agents (phosphate from the PBS buffer, thiol/amine/carboxylate functions from desorbed penicillamine ligands) can accelerate the dissolution process, and second, the generation of reactive oxygen species (ROS) is favored in the presence of QDs and O_2_ under light irradiation [33]. For longer irradiation times (2 h), also in the case of the thin and thick shell QDs, the QD core rapidly degrades to form In–phosphate and In–carboxylate secondary products. The formation of Se and Zn secondary products occurs later, mainly after 6 h and 24 h, respectively. The thick shell QDs are more resistant to degradation than the thin shell QDs, with some fluorescence remaining after 90 min of aging. 

We previously showed that these InP QDs are safe in their pristine form, but show significant cytotoxicity and genotoxicity after aging [18]. Aged QDs impair the viability, oxidative balance, and DNA integrity in exposed cells, but also the overall cellular metal homeostasis [18]. The latter can be attributed to the release of In, Zn, and Se ions from degraded QDs. Overall, these results suggest that aged InP QD toxicity is triggered by In(III)-phosphate, In(III)-carboxylate, and Zn(II)-phosphate. Therefore, in order to develop even safer InP QDs, one would need to reduce, as much as possible, the release of such In(III) and Zn(II) forms.

## 5. Conclusions

We report the progressive degradation of multiple shell InZnP QDs upon exposure to simulated solar light under environmentally relevant conditions, with a gradual loss of their optical properties, i.e., their typical excitonic peak, their bright photoluminescence, and of their crystalline structure. Typically, their PL reaches 10% of its initial value after less than 15 min, 60 min, and more than 90 min for gradient shell, thin shell, and thick shell QD, respectively. The transformation products that form when they degrade are indium–phosphate and indium–carboxylate precipitates, as well as zinc–phosphate precipitates and reduced forms of selenium, both red Se(0) and selenite. QDs with a thick ZnS outer shell show the best resistance to photodegradation. While pristine QDs are not toxic, aged QDs show significant cytotoxicity, with LD50 values of 77 nM for pristine gradient shell QD while all three aged QD samples show a DL50 ranging from 15 to 23 nM. Therefore, QDs with a thick outer shell could be considered as a safer-by-design alternative to the gradient shell and thin shell QDs, because they resist, more than the others, to photodegradation as well as showing better photoluminescence properties, with photoluminescence quantum yields of 24% compared to 18% for gradient shell and thin shell QDs. Still, although more resistant, these QDs tend to degrade when exposed in environmental conditions in the presence of complexing agents such as phosphates from the PBS buffer. Zinc chalcogenide shells are prone to oxidative degradation processes and the use of more robust outer shell materials (e.g., composed of other metal oxides) can be proposed if high environmental stability and lower toxicity of QDs is targeted.

## Figures and Tables

**Table 1:** Structural parameters for In, Se and Zn atoms determined by shell fitting, and comparison with some reference compounds.

		R (Å)	*n*	σ^2^ (Å^2^)		R (Å)	*n*	σ^2^ (Å^2^)		R (Å)	*n*	σ^2^ (Å^2^)		R (Å)	*n*	σ^2^ (Å^2^)		R (Å)	*n*	σ^2^ (Å^2^)	RF
In speciation																					
Grad pristine	P	2.53	4.2	0.004	Se	2.75	1.5	0.003													0.002
Thin pristine	P	2.53	4.1	0.0046	Se	2.57	1.9	0.0046													0.003
Thick pristine	P	2.53	4.2	0.0044	Se	2.56	1.3	0.0044													0.005
InP (crys.) [25]	P	2.53	4	0.0022																	
InSe (am.) [26]	Se	2.63	3	0.0075																	
In_4_Se_3_ (crys.) [28]	Se	2.80	2	0.0064																	
Zn speciation																					
Grad pristine	S	2.34	2.6	0.0052 ^§^	Se	2.46	1.5	0.005 ^§^	Zn	3.88	4.3	0.012 ^#^	Zn	4.47	2.0	0.0120 ^#^	S	4.46	2.6	0.012 ^#^	0.008
Thin pristine	S	2.33	3.0	0.0068 ^§^	Se	2.44	1.4	0.006 ^§^	Zn	3.85	4.0	0.012 ^#^	Zn	4.40	1.5	0.0120 ^#^	S	4.40	1.7	0.012 ^#^	0.013
ZnS [29]	S	2.34	4.0										Zn	4.48	12.0						
ZnSe [30]					Se	2.45	4.0		Zn	4.00	12.0										
Se speciation																					
Grad pristine	Zn	2.46	4.0	0.004																	0.009
Thin pristine	Zn	2.46	4.0	0.0041																	0.030
Thick pristine	Zn	2.46	4.0	0.0048																	0.051
ZnSe [30]	Se	2.45	4.0																		

*n*: number of atoms, R: interatomic distance, σ^2^: Debye-Waller factor, RF: R-factor, i.e., residual between fit and experiment. For In, range of the fit in k space: 3.4–12.9 Å^−1^, R range: 1.2–2.7 Å. In-P and In-Se paths were calculated using FEFF code, based on the structure of InP and InSe. For Zn, range of the fit in the k space: 4.3–14.3 Å^−1^, R range: 1.3–2.7 Å. Paths were calculated based on the structure of ZnS and ZnSe. The σ^2^: values were correlated during adjustment for the two first shells (^§^) and the three last ones (^#^). For Se, range of the fits in the k space: 3.6–12.5 Å^−1^, R range: 1.0–4.5 Å. Paths were calculated based on the structure of ZnSe.

## Data Availability

Data can be accessible upon request to the corresponding author.

## Supplementary Materials

The following supporting information can be downloaded at: https://www.mdpi.com/article/10.3390/nano12203703/s1, Table S1: Photophysical and structural properties of the used QDs after aqueous phase transfer; Table S2: Results of the linear combination fits of XANES and EXAFS spectra for the aged QDs; Figure S1: Photophysical properties of QDs after aqueous phase transfer; Figure S2: Photophysical transformation of the three QDs during the aging process in a climatic chamber; Figure S3: Pictured of aged QDs; Figure S4: Structural characterization of aged gradient shell QD by STEM-EDX analysis; Figure S5: Structural characterization of aged thin shell QD by STEM-EDX analysis; Figure S6: Structural characterization of thin shell QD, aged in water, by STEM-EDX analysis; Figure S7: In K-edge EXAFS analysis of pristine and aged QDs; Figure S8: Indium K-edge analysis of thin shell and thick shell QDs; Figure S9: In K-edge XANES spectra for the pristine and aged QDs; Figure S10: Zn K-edge EXAFS results; Figure S11: Se K-edge XANES and EXAFS results. References [17,18] are cited in the supplementary materials.
