# Supplementary material for: Physico-Chemical Transformation and Toxicity of Multi-Shell InP Quantum Dots under Simulated Sunlight Irradiation, in an Environmentally Realistic Scenario
Source: Nanomaterials (Basel). 2022 Oct 21;12(20):3703. doi: 10.3390/nano12203703 (PMC9611924; doi:10.3390/nano12203703)
Supplement: Supplementary file 1 [file nanomaterials-12-03703-s001.zip › nanomaterials-1973011-SI.pdf]

Supplementary information for:

Physico-Chemical Transformation and Toxicity of Multi-Shell InP Quantum Dots under Simulated Sunlight Irradiation, in an Environmentally Realistic Scenario

Fanny Dussert [1], Géraldine Sarret [2], Karl David Wegner [3], Olivier Proux [4], Gautier Landrot [5], Pierre-Henry Jouneau [6], Peter Reiss [3] and Marie Carrière [1,*]

[1] University Grenoble Alpes, CEA, CNRS, IRIG-SyMMES, CIBEST, 38000 Grenoble, France
[2] University Grenoble Alpes, University Savoie Mont Blanc, CNRS, IRD, IFSTTAR, ISTerre, 38000 Grenoble, France
[3] University Grenoble Alpes, CEA, CNRS, IRIG-SyMMES, STEP, 38000 Grenoble, France
[4] University Grenoble Alpes, CNRS, IRD, Météo-France, INRAE, Observatoire des Sciences de l'Univers de Grenoble (OSUG), UAR 832 CNRS, 38400 Saint Martin d'Hères, France
[5] Synchrotron SOLEIL, L'Orme des Merisiers, 91190 Saint Aubin, France
[6] University Grenoble Alpes, CEA, CNRS, IRIG-MEM, LEMMA, 38000 Grenoble, France
* Correspondence: marie.carriere@cea.fr; Tel.: +33-438-780328

Table S1. Photophysical and structural properties of the used QDs after aqueous phase transfer (UV-vis and PL spectra: cf. Figure S1)

	Gradient shell	Thin shell	Thick shell
1st exciton peak (nm)	518	512	518
PL maxima	571	562	568
FWHM	59.7	56.8	61
PLQY	18%	18%	24%
Indium (%at.)	1	1	1
Phosphorus (%at.)	1.1	0.9	1
Zinc (%at)	1.9	5.1	8.6
Selenium (%at)	0.5	1	1.2
Sulfur (%at)	2	5	9.1
Core diameter	2.67±0.32 nm	2.67±0.32 nm	2.67±0.32 nm
Core-shell diameter	3.6±1.0 nm	4.1±0.8 nm	6.2±1.0 nm
Nr. of shell layers	2 monolayers	3 monolayers	4-5 monolayers
Z-average, growth medium (PdI)	565 nm (0.645)	151 nm (0.346)	467 nm (0.820)
Zeta potential in PBS	-21 mV	-22 mV	-21 mV

These data have been published previously [1,2]. PL: photoluminescence. PLQY: photoluminescence quantum yield. Core and core-shell diameters were measured from cryo-TEM images, acquired on a FEI Polara microscope operating at 300 kV and recorded on a Gatan K2 camera. Number of shell layers (Nr. shell layers) was deduced from EDX data. DLS and zeta potential measurements were recorded on a zetasizer Nano Series (Malvern).

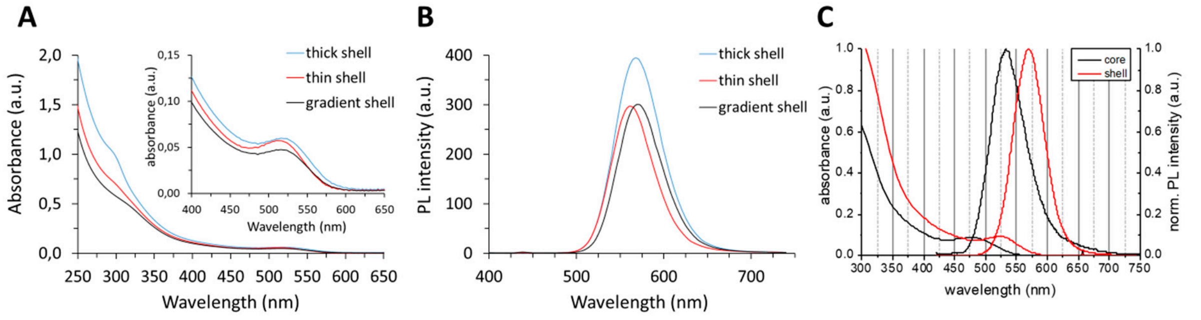

Figure S1. Photophysical properties of QDs after aqueous phase transfer. UV-vis absorbance (**A**) and photoluminescence (**B**) spectra (excitation wavelength: 400 nm). Inset in (A) is rescaled view in the 400-650 wavelength range, where the excitonic peak appears. The absorbance increase in the 270-320 nm range is indicative of successful ZnS shell growth. (**C**) UV-vis absorbance and photoluminescence spectra of core only (core) and gradient shell (shell) InP QDs, showing the 40-50 nm red shift due to gradient shell growth on the core (note that these spectra were acquired on another batch of QDs, explaining why the excitonic peak and maximum PL values slightly differ in (C), as compared to (A) and (B)).

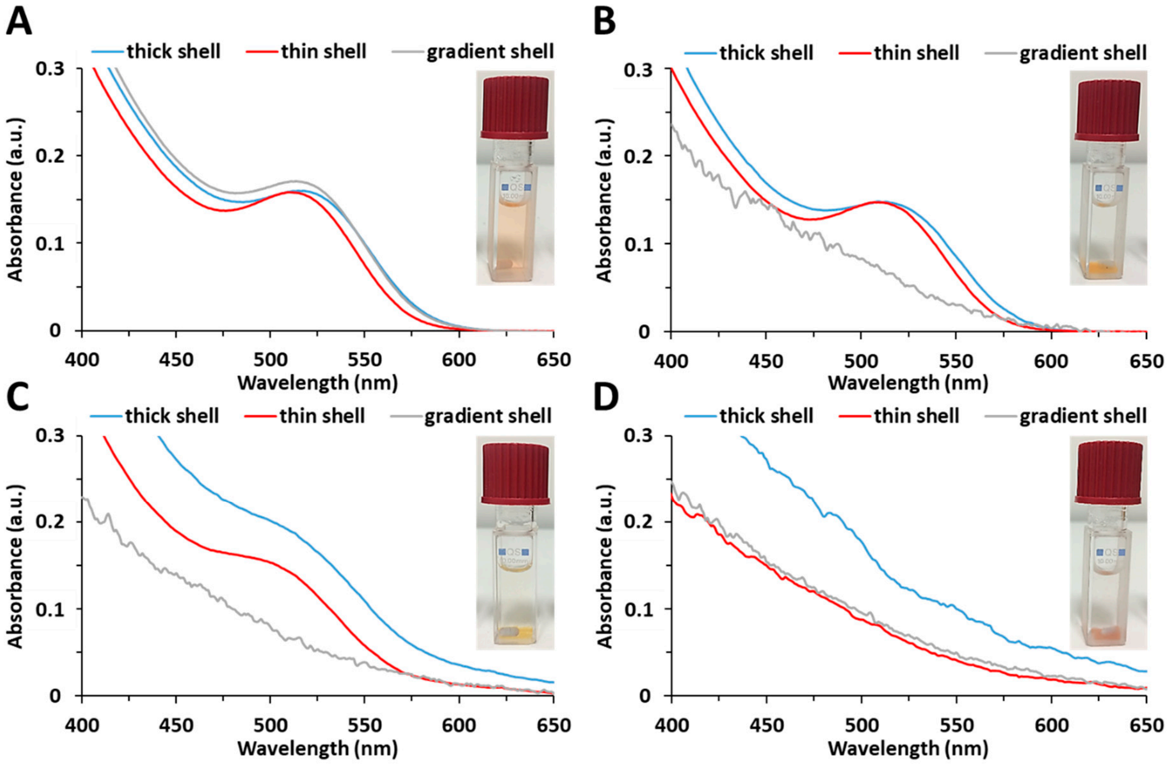

Figure S2. Photophysical transformation of the three QDs during the aging process in a climatic chamber. UV-vis absorbance spectra of pristine QDs (**A**), or after 15 min (**B**), 30 min (**C**) and 45 min of aging (**D**). Insets are optical images of the thick shell QD at the respective time of aging, showing the macroscopic changes in QD suspensions during the aging process: QDs were initially stable colloidal suspensions with orange color (A, inset). These suspensions first precipitated as orange deposits after 15 min of ageing (B, inset), then the precipitates lightened and became yellow after 30 min of ageing (C, inset), then their colour became pink after 45 min of ageing (D, inset).

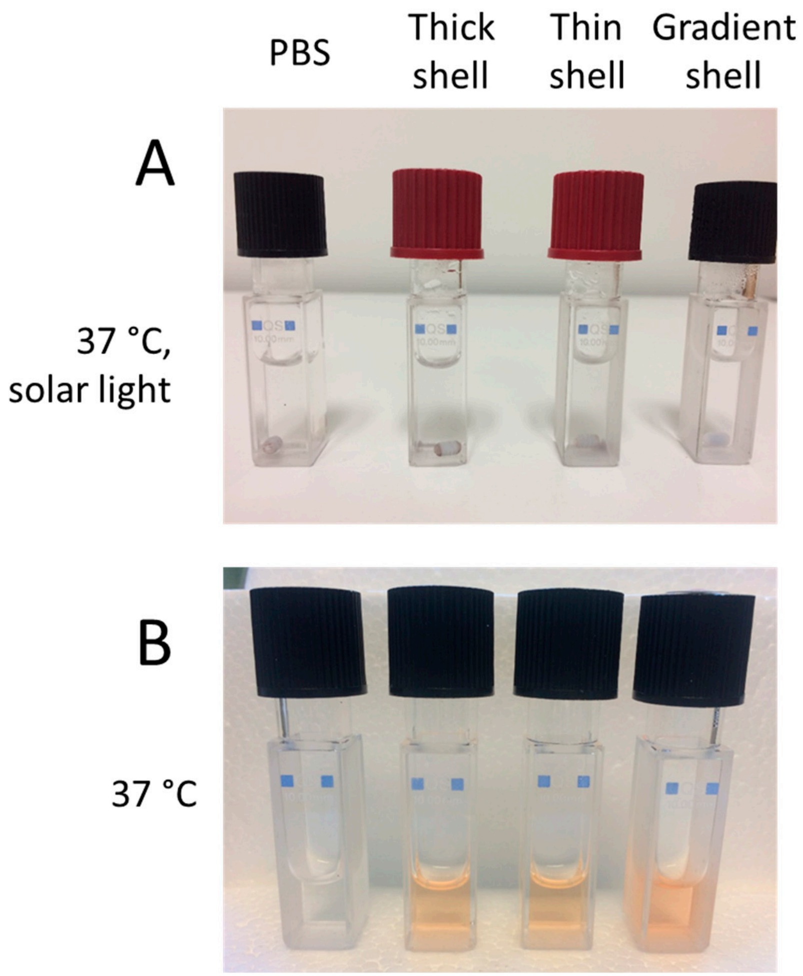

Figure S3. Pictures of aged QDs. QDs were exposed for 24 h either to increased temperature with no light (37°C) (**A**) or to increased temperature (37°C) under irradiation with simulated solar light (**B**), proving that irradiation is responsible for the observed QD degradation.

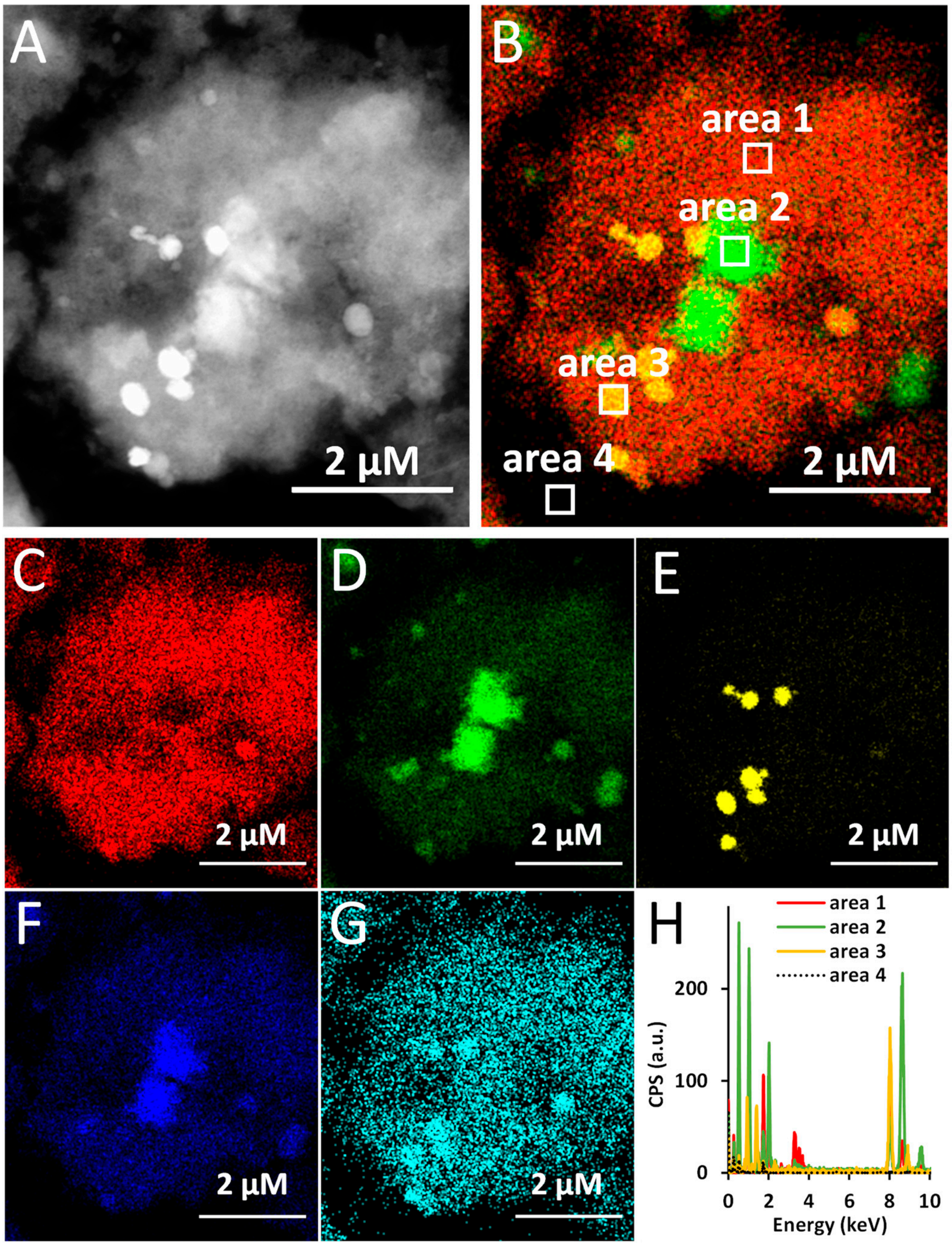

Figure S4. Structural characterization of aged gradient shell QD by STEM-EDX analysis. The gradient shell QD was aged in PBS. STEM (HAADF) imaging (**A**) and EDX imaging and analysis (**B-H**). B is the merge of the distribution of In, Zn and Se (also represented in C, D and E, respectively) in the sample. Distributions of P and S (F and G, respectively), as well as EDX spectra recorded from areas 1, 2, 3 and 4 from B (H).

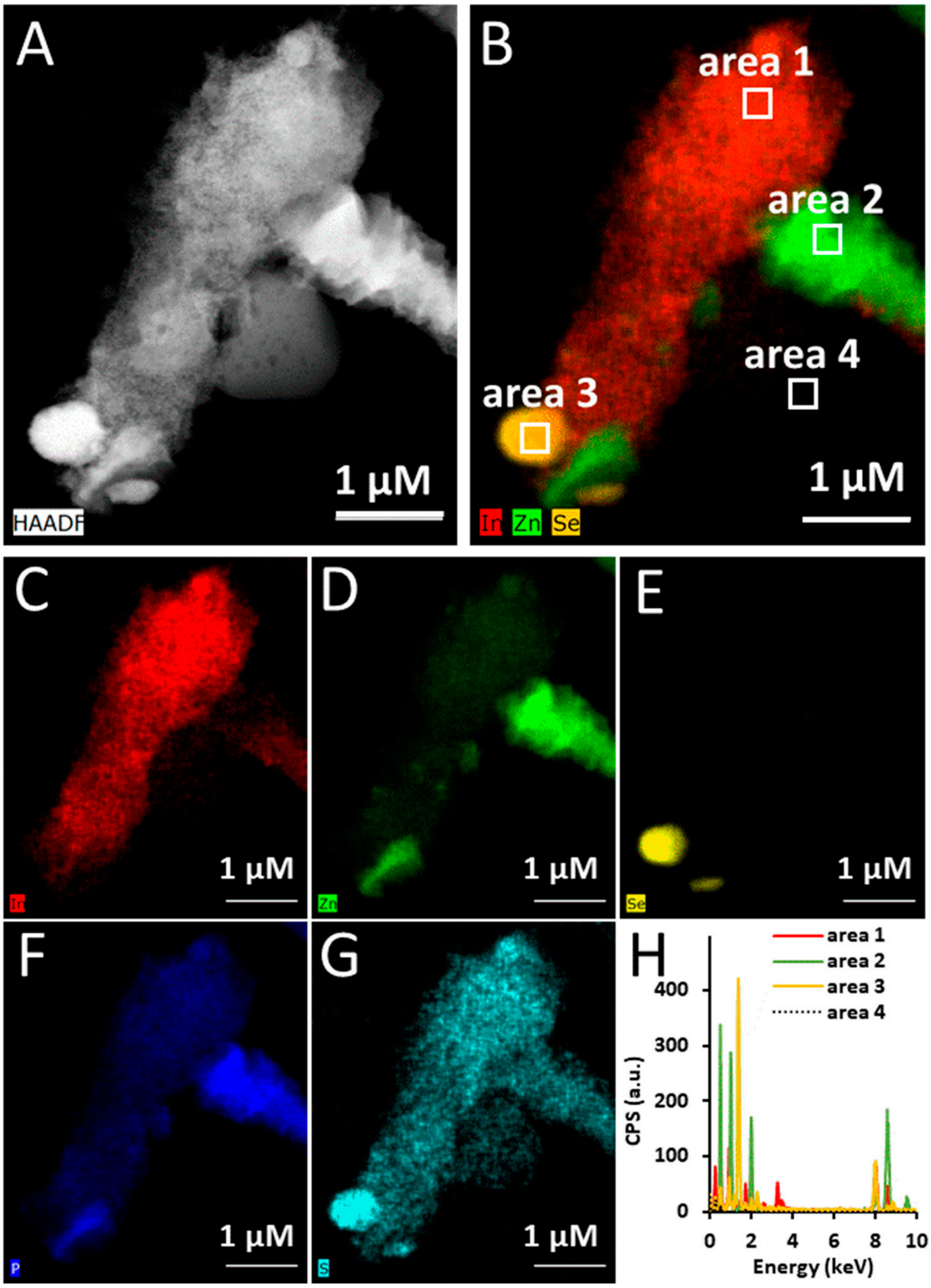

Figure S5. Structural characterization of aged thin shell QD by STEM-EDX analysis. The gradient thin QD was aged in PBS. STEM (HAADF) image (**A**) and EDX analysis and imaging (**B-H**). B is the merge of the distribution of In, Zn and Se (also represented in C, D and E, respectively) in the sample. Distributions of P and S (F and G, respectively) in the sample are also represented, as well as EDX spectra recorded from areas 1, 2, 3 and 4 from B (H).

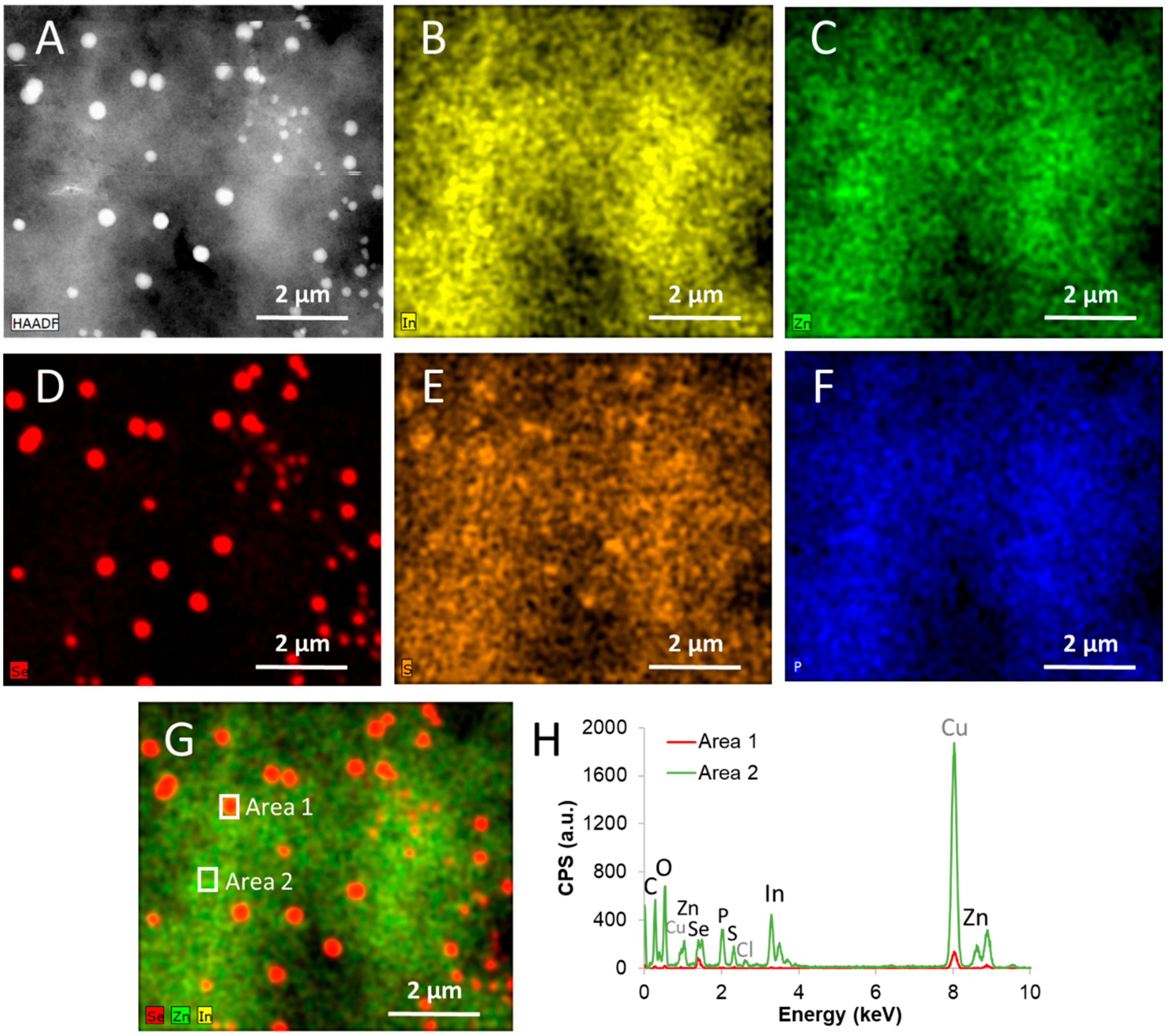

Figure S6. Structural characterization of thin shell QD, aged in water, by STEM-EDX analysis. STEM (HAADF) image (**A**) and EDX imaging and analysis (**B-H**). In (**B**), Zn (**C**), Se (**D**), S (**E**), P (**F**) elemental distributions. Merge of In, Zn and Se distributions (**G**), and location of Areas 1 and 2 whose EDX spectra are represented in (**H**).

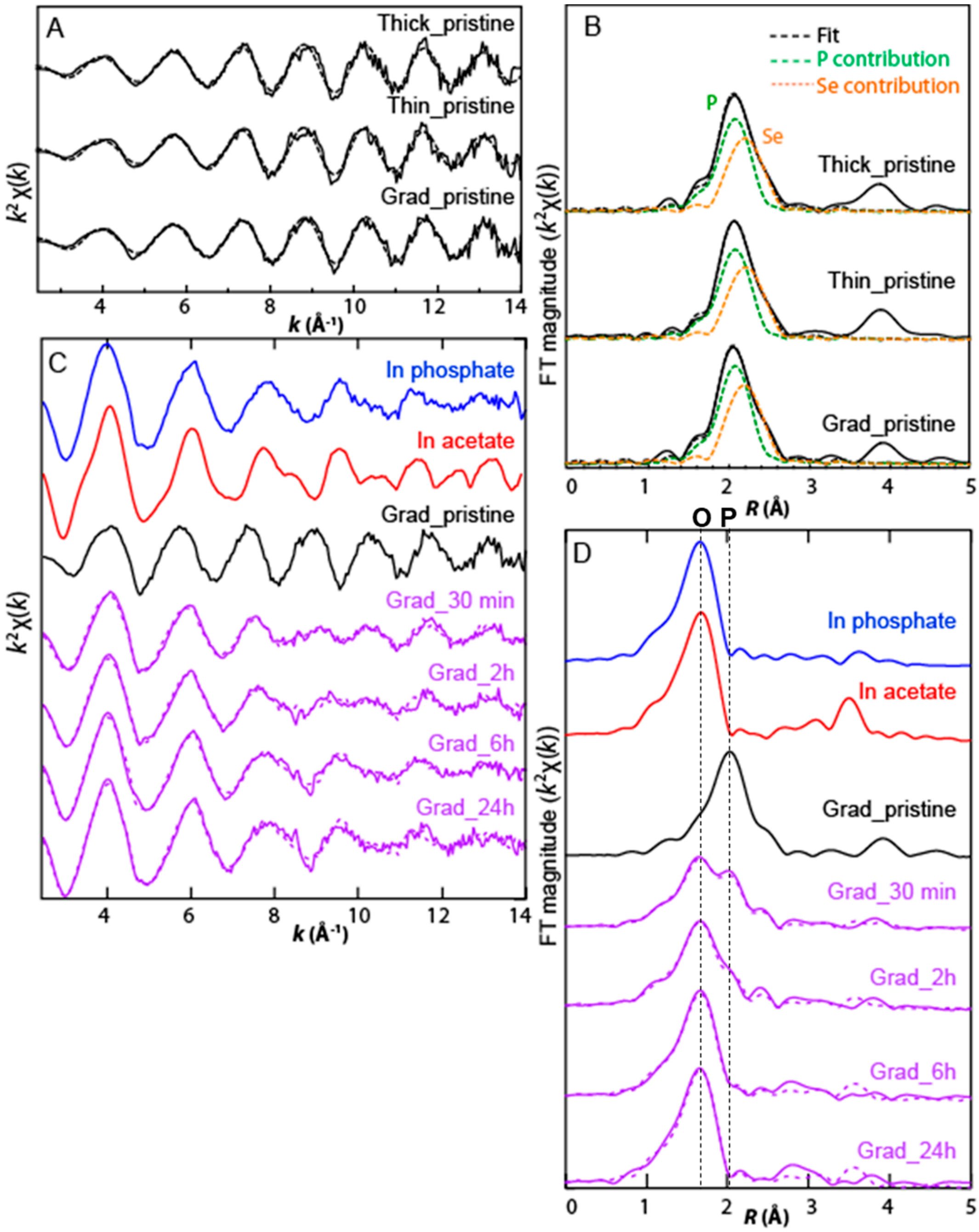

Figure S7. In K-edge EXAFS analysis of pristine and aged QDs. EXAFS (**A**) and Fourier transformed (**B**) spectra of pristine QDs. EXAFS (**C**) and Fourier transformed (**D**) spectra recorded for the gradient shell QD at different aging time-points. Experimental data are represented as solid lines and first shell fits as dashed lines, showing that the fit qualities are good. The contribution of In-P and In-Se coordinations in Fourier-transformed EXAFS spectra are illustrated in (B) ("P" for In-P and "Se" for In-Se). The contributions of In-P and In-O coordinations are illustrated in (D) ("P" for In-P and "O" for In-O, as well as dashed vertical lines showing the peak positions). Deduced structural parameters are reported in Table 1.

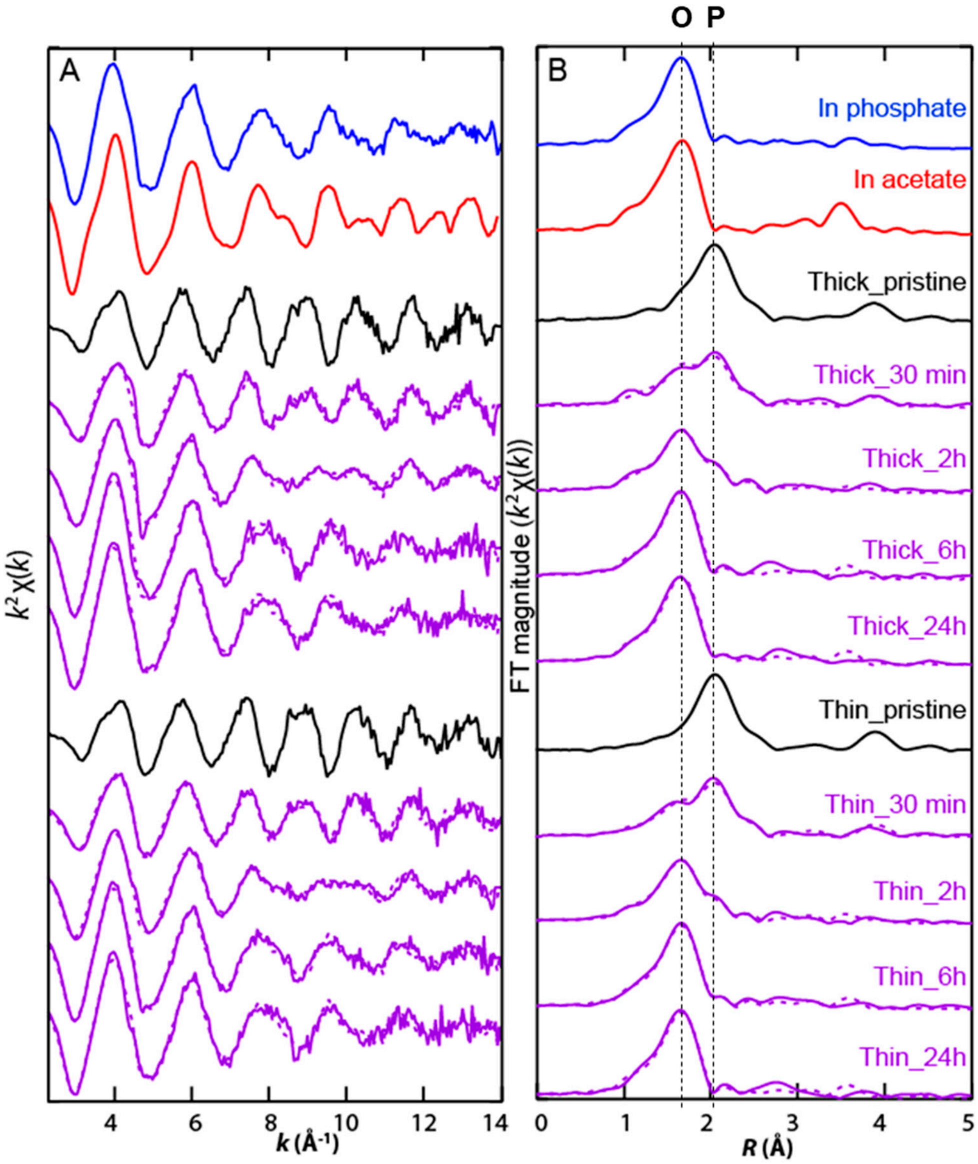

Figure S8. Indium K-edge analysis of thin shell and thick shell QDs. K-edge EXAFS (**A**) and Fourier transformed (**B**) spectra for the thick and thin QDs at different time-points of aging. Experimental data (solid lines) and linear combination fits (dashed lines), showing the good fit qualities. The contributions of In-P and In-O coordinations are illustrated in (B) ("P" for In-P and "O" for In-O, as well as dashed vertical lines showing the peak positions). Deduced structural parameters are reported in Table 1.

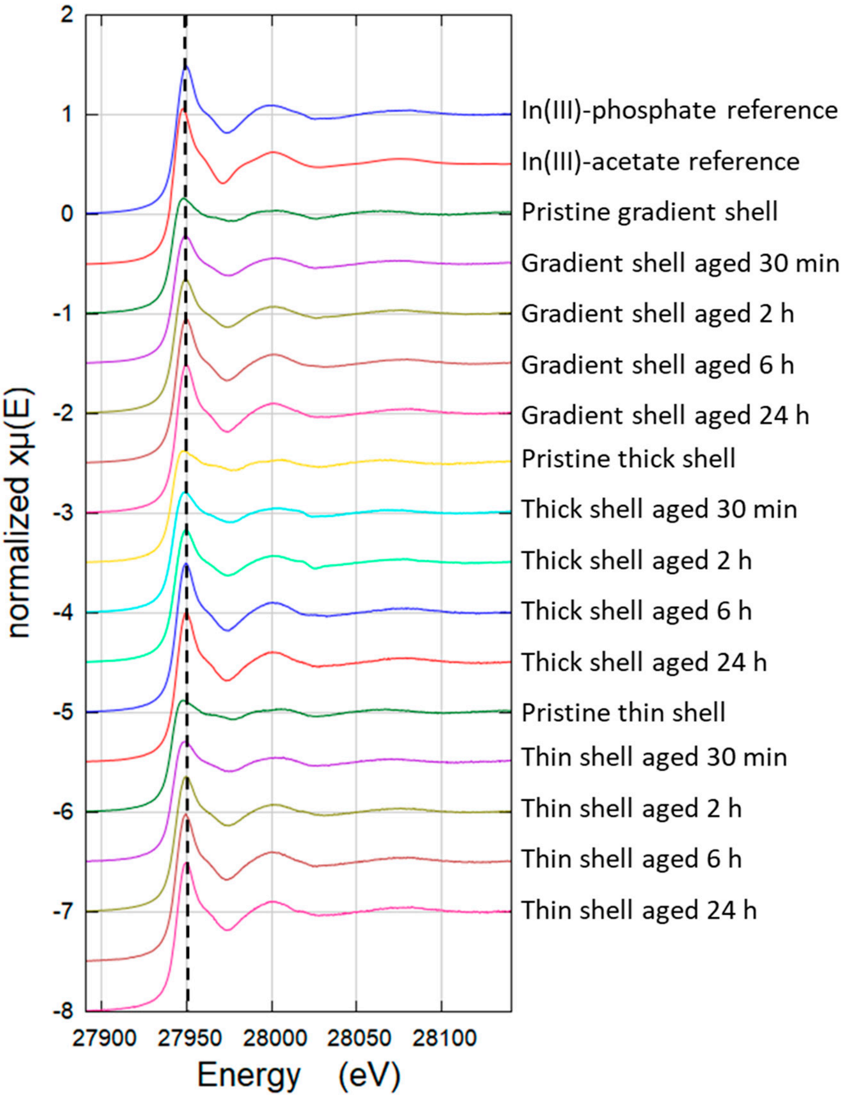

Figure S9. In K-edge XANES spectra for the pristine and aged QDs. The maximum of the absorption edge remains at 27.95 keV (dashed line), proving that the oxidation state of indium is +III in both pristine and aged QDs.

Table S2. Results of the linear combination fits of XANES and EXAFS spectra for the aged QDs[a]

	Fit region	Prist. QD	In-acet	In-phos	Zn-phos	Red Se(0)	Se(IV)	Sum	R-factor
In species									
Thin 24 h	EXAFS	0	19	75				94	0.047
Thin 6 h	EXAFS	8	14	76				98	0.025
Thin 2 h	EXAFS	27	20	44				91	0.041
Thin 30 min	EXAFS	70	5	20				95	0.062
Thick 24 h	EXAFS	0	26	71				97	0.030
Thick 6 h	EXAFS	0	22	72				94	0.047
Thick 2 h	EXAFS	33	11	51				95	0.039
Thick 30 min	EXAFS	64	6	25				93	0.071
Grad 24 h	EXAFS	0	29	64				93	0.038
Grad 6 h	EXAFS	7	22	61				90	0.031
Grad 2 h	EXAFS	30	14	49				93	0.042
Grad 30 min	EXAFS	47	11	34				92	0.039
Zn species									
Thin 24 h	EXAFS	0			102			102	0.046
Thin 2 h	EXAFS	88			13			101	0.060
Thin 30 min	EXAFS	88			13			101	0.053
Grad 24 h	EXAFS	0			106			106	0.085
Grad 2 h	EXAFS	81			18			99	0.056
Grad 30 min	EXAFS	91			13			104	0.067
Se species									
Thin 24 h	XANES					71	27	98	0.005
Thin 6 h	XANES	53				30	16	99	0.003
Thin 30 min	XANES	97					3	100	0.001
Thick 24 h	XANES					67	33	100	0.009
Thick 2 h	XANES	97					3	100	0.001
Thick 30 min	XANES	99						99	0.001
Grad 24 h	XANES	22				77	4	100	0.001
Grad 6 h	XANES	61				39		100	0.001
Grad 30 min	XANES	94					5	99	0.001

[a]R-Factor: residual between fit and experimental data. Prist.: pristine. In-acet: In-acetate. In-phos: In-phosphate

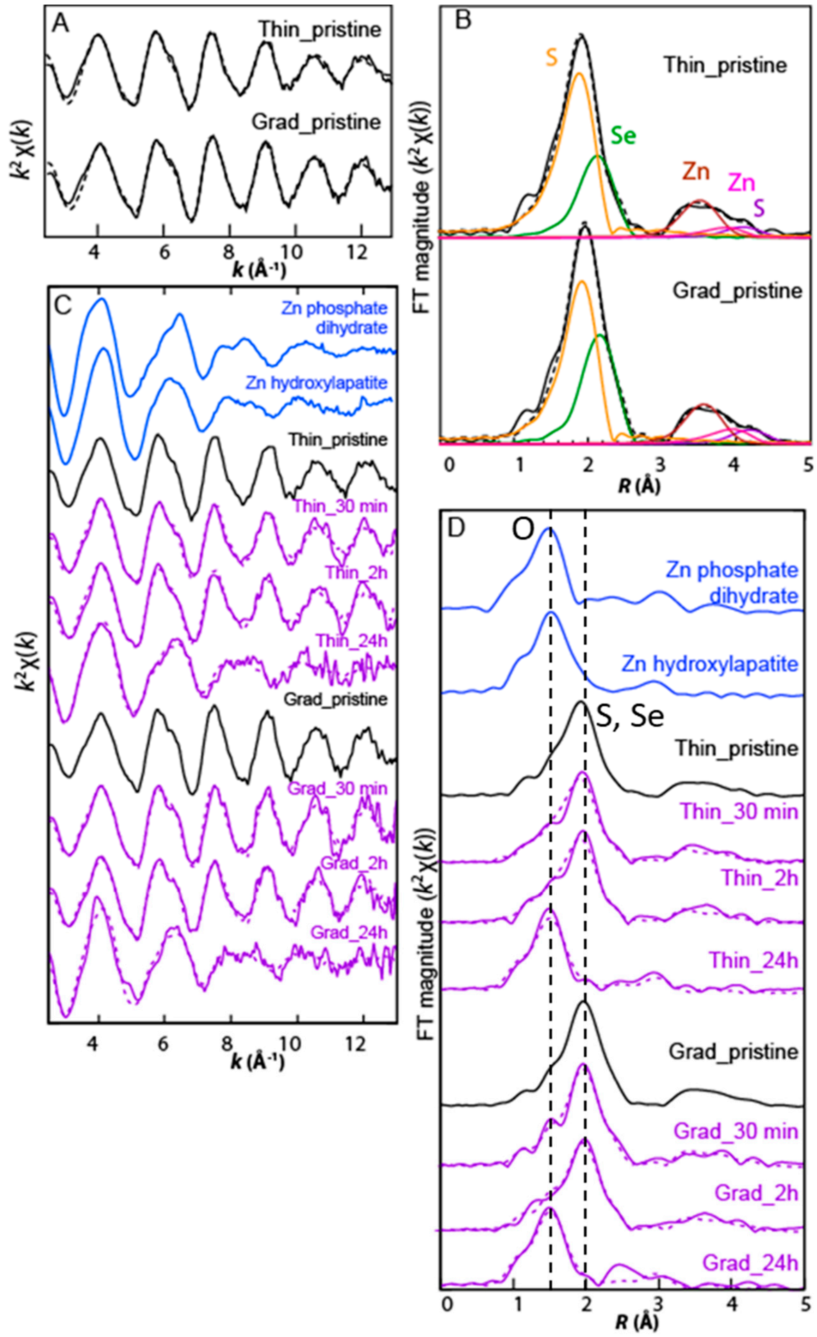

Figure S10. Zn K-edge EXAFS results. EXAFS (**A**) and Fourier transformed (**B**) spectra for the pristine QDs, and shell fits (dashed lines, structural parameters given Table 1), showing the good fit qualities. EXAFS (**C**) and Fourier transformed (**D**) spectra for the QDs at different aging time-points, and their linear combination fits (dashed lines). The contributions of Zn-S, Zn-Se, Zn-Zn and Zn-O coordinations are illustrated in (B) and (D) ("S" for Zn-S, "Se" for Zn-Se, or "Se,S" as both these contributions cannot be discriminated in (D), "Zn" for Zn-Zn and "O" for Zn-O, as well as dashed vertical lines showing the peak positions in (D). Deduced structural parameters are reported in Table 1.

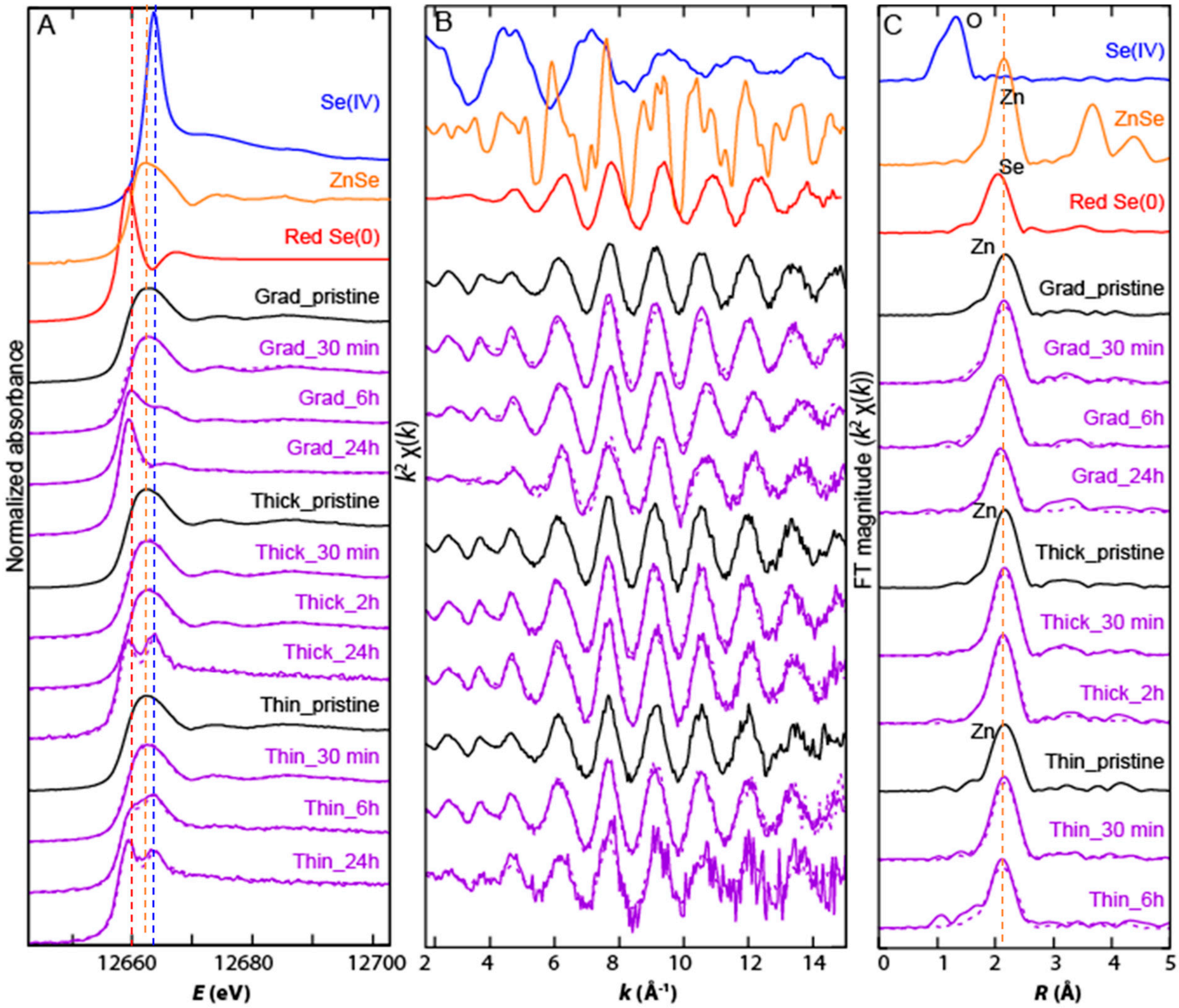

Figure S11. Se K-edge XANES and EXAFS results. XANES (**A**), EXAFS (**B**) and Fourier transformed (**C**) spectra for the pristine and aged QDs (solid lines), and LCFs using the pristine QDs, red Se(0) and Na-selenite as components (dashed lines in (B) and (C)). XANES spectra (A) show the shifts of the absorption edge around its position in pristine QDs, i.e., 12.662 keV (orange dashed line), evidencing oxidation of Se to Se(0) at 12.660 keV (red dashed line) and Se(IV) at 12.663 keV (blue dashed line) as QDs are aged. (B) shows the good quality of EXAFS fits and highlights that the spectra recorded from QD samples aged for 24 h are too noisy to be analysed properly. Fourier-transformed EXAFS spectra (C) show the position of the contributions of Se-O, Se-Zn, Se-Se coordinations, highlighting that the main contributor atom in the structure of both pristine and aged QDs is the Se-Zn coordination (orange dashed line in (C)).

References

1. F. Dussert, K. D. Wegner, C. Moriscot, B. Gallet, P. H. Jouneau, P. Reiss and M. Carriere, Evaluation of the dermal toxicity of InZnP quantum dots before and after accelerated weathering: toward a safer-by-design strategy, *Frontiers in Toxicology*, 2021, **3**, 636976.
2. K. D. Wegner, F. Dussert, D. Truffier-Boutry, A. Benayad, D. Beal, L. Mattera, W. L. Ling, M. Carrière and P. Reiss, Influence of the Core/Shell Structure of Indium Phosphide Based Quantum Dots on Their Photostability and Cytotoxicity, *Frontiers in chemistry*, 2019, **7**, 466.
